# Brachytherapy and anterior segment imaging in iris melanoma

**DOI:** 10.3332/ecancer.2017.734

**Published:** 2017-04-18

**Authors:** Jin Soo Andy Song, Adam A Dmytriw, Hesham Lakosha

**Affiliations:** 1 Department of Ophthalmology and Vision Sciences, Dalhousie Medical School, 6299 South St, Halifax, NS B3H 4R2, Canada; 2 Department of Medical Imaging, University of Toronto, 263 McCaul St, Toronto, ON M5T 1W7, Canada

**Keywords:** melanoma, brachytherapy, ocular, oncology, ultrasound

## Abstract

A 40-year-old male presented to the ophthalmology clinic with a darkly pigmented infratemporal lesion in his right eye. The corrected visual acuity in both eyes was 6/6 and both pupils were equal and reactive. Slit lamp biomicroscopy showed a well-demarcated and heavily pigmented lesion in the peripheral iris between 6 and 8 o’clock. Ultrasound biomicroscopy (UBM) revealed a solid mass deriving from the iris stroma without ciliary body involvement, helping to classify the uveal melanoma and establishing the diagnosis of iris melanoma. Fine needle aspiration (FNA) confirmed melanoma with inactivation of the BAP1 gene. The patient was treated with brachytherapy using an I-125 plaque. Follow-up UBM, three years later, demonstrated significantly reduced dimensions of the tumour. UBM has become crucial to the differentiation of uveal melanomas from benign growths, and lesions <3 mm cannot be reliably visualised by other imaging modalities or localised to the correct uveal structure. Brachytherapy represents a safe and effective treatment option even in lesions that are BAP1 positive.

## Introduction

Uveal melanomas are the most common primary intraocular tumour in adults (an annual incidence of 5.1 per million) of which iris melanomas account for 3%–10% [3]. Risk factors for uveal melanomas are predominantly non-modifiable, and include Caucasian descent, lighter iris colour, and inactivation of the BAP1 gene occurring in 84% of metastasising tumours [4].

## Case presentation

**Figure 1.**
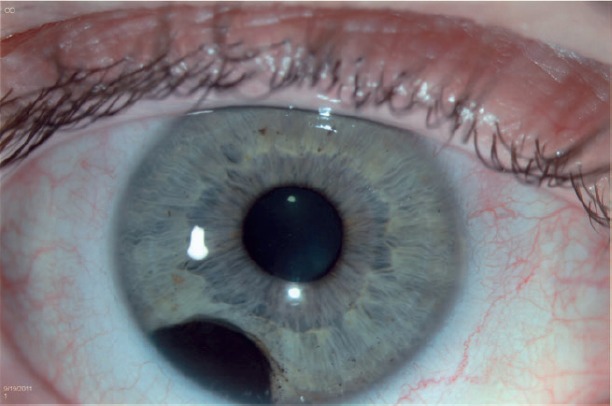
The heavily pigmented infratemporal lesion on clinical exam was shown by slit lamp biomicroscopy to represent a well-demarcated within the peripheral iris between 6 and 8 o’clock.

**Figure 2.**
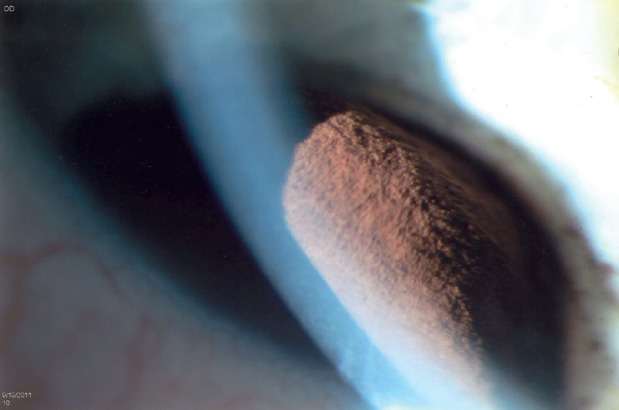
The largest basal diameter was 4 mm × 2.5 mm, one of the most important features for estimating prognosis.

A 40-year-old asymptomatic non-smoking Caucasian male with no past ocular history presented with a darkly pigmented infratemporal lesion in his right eye first noticed one year prior. The corrected visual acuity in both eyes was 6/6, measured intraocular pressure (IOP) was 15 and 16 mmHg in the right and left eyes respectively, and both the pupils were equal and reactive. Slit lamp biomicroscopy showed a well-demarcated and heavily pigmented lesion in the peripheral iris between 6 and 8 o’clock (Figure 1). Fundus examination was normal, and gonioscopic examination revealed total angle closure by the tumour for two hours. The estimated basal diameter for this elevated lesion measured at 4 mm × 2.5 mm (Figure 2).

**Figure 3.**
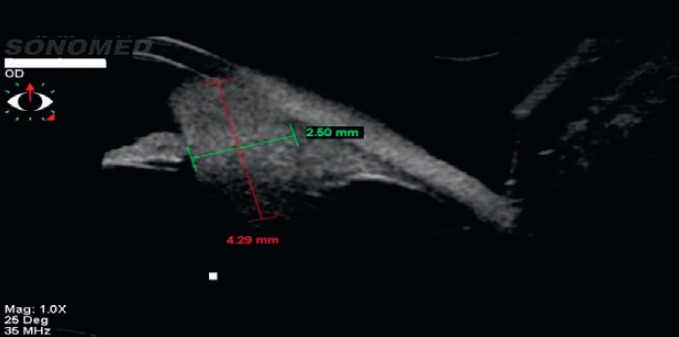
UBM showed a solid mass with high/medium reflectivity and loss of the posterior iris plane. The mass appears to derive from the iris stroma without definite ciliary body involvement.

**Figure 4.**
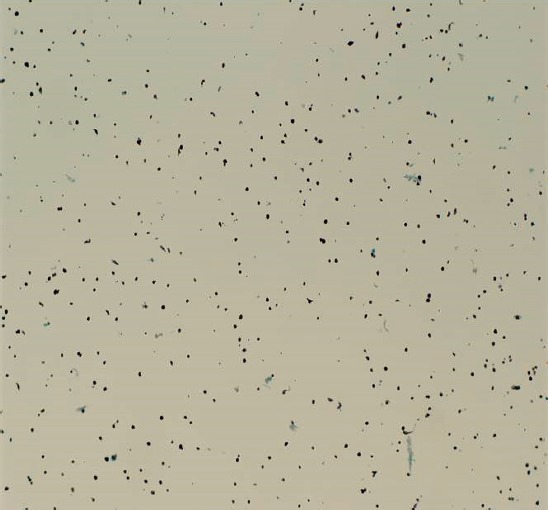
Low-power microscopy of cellular aspirate, demonstrating satisfactory yield of melanotic cells.

**Figure 5.**
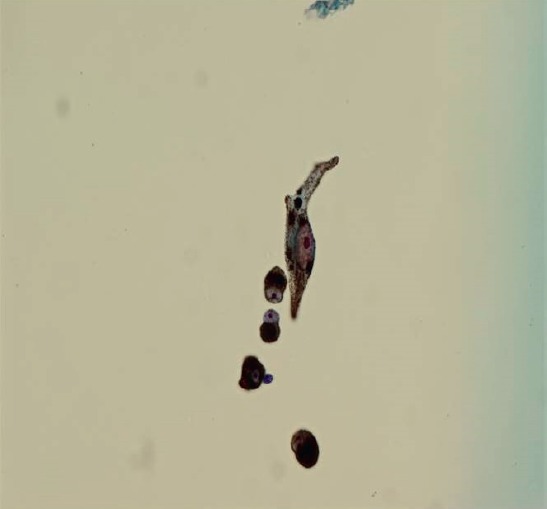
High magnification microscopy, showing spindle cell with a large nucleus and prominent nucleoli. Large melanosomes confirm spindle B melanoma cells.

Ultrasound biomicroscopy (UBM) revealed a solid mass deriving from the iris stroma without ciliary body involvement, suggesting a diagnosis of iris melanoma (Figure 3). FNA confirmed melanoma with a mix of oval spindle and polyhedral epithelioid cells, and an inactivation of the BAP1 gene (Figures 4 and 5). Both the findings increased the likelihood of fatal metastasis, and thus closer surveillance was indicated [1].

The patient was treated with brachytherapy using a 12-mm diameter I-125 plaque implanted over the ciliary body and iris at the 7’o clock meridian for one week for a total dose of 82 Gy.

**Figure 6.**
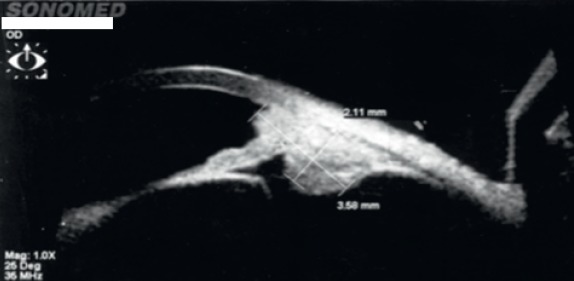
UBM at three-year post-brachytherapy shows reduced tumour dimensions. Peripheral tumour location allowed for a treatment safety margin with a favourable visual prognosis.

UBM, three years later, demonstrated the success of radiotherapy through the significantly reduced dimensions of the tumour (Figure 6). Following radiotherapy, the patient developed a cataract, the most common complication occurring in 83% of cases [2].

## Discussion

Malignancies without BAP1 inactivation generally have a favourable prognosis because of their lower rates of visual morbidity and metastasis and a five-year mortality rate of 2%–3% [3]. Hence, management should reflect these auspicious outcomes. As the area of treatment involves a safety margin beyond the borders of the tumour, the effects of brachytherapy on visual prognosis is often dependent on the proximity of the tumour from the fundus. Given the peripheral placement of this patient’s tumour, he had a favourable prognosis. The American Brachytherapy Society states an average follow-up of 53 months, with most eyes examined every six months and modulated based on the likelihood of secondary complications, notably a shortened interval for posterior tumours because of the higher risk of radiation maculopathy and optic neuropathy [5].

UBM has become crucial to the differentiation of uveal melanomas from benign growths, as lesions <3 mm cannot be reliably visualised by other imaging modalities [6]. As these cancers are often asymptomatic and indolent, careful monitoring is imperative to determine whether it is necessary to subject a patient to invasive biopsy or possible complications from surgical intervention or radiology. Advantages of UBM in anterior segment imaging include visualisation of structures inaccessible to conventional methods, such as the ciliary body and zonules, illustrating the degree of penetration of iris root and ciliary face in delineating solid masses from cystic lesions and recording high-resolution cross-sectional images of diameter and thickness to document growth and determine malignancy [7]. The higher resolution afforded by UBM specifies the size, boundary, and elevation of the tumour, which are important parameters in predicting metastasis and mortality. Additional findings consolidating the diagnosis include pronounced vasculature, hyphema, secondary cataract or glaucoma, and most importantly documented growth [1].

Clinicians should have a low threshold to refer or work up cases of pigmented eye uveal lesions because of their similarity in appearance of nevus and melanoma, and UBM acts as an effective modality to elucidate a diagnosis in these ambiguous cases. The differential diagnosis for uveal lesions includes iris nevus, iris cyst, essential iris atrophy, and foreign body. In differentiating iris melanoma from nevus on UBM, common findings for melanoma include medium–high reflectivity, possible cavitation, and, most importantly, a loss of the posterior iris plane [7].
